# Lack of transmission of *Sugarcane yellow leaf virus* in Florida from Columbus grass and sugarcane to sugarcane with aphids or mites

**DOI:** 10.1371/journal.pone.0230066

**Published:** 2020-03-06

**Authors:** Wardatou Boukari, Chunyan Wei, Lihua Tang, Martha Hincapie, Moramay Naranjo, Gregg Nuessly, Julien Beuzelin, Sushma Sood, Philippe Rott

**Affiliations:** 1 Department of Plant Pathology, Everglades Research and Education Center, University of Florida, Belle Glade, Florida, United States of America; 2 State Key Laboratory for Conservation and Utilization of Subtropical Agro-Bioresources, College of Agriculture, Guangxi University, Nanning, China; 3 Florida Crystals Corporation, Belle Glade, Florida, United States of America; 4 Department of Entomology and Nematology, Everglades Research and Education Center, University of Florida, Belle Glade, Florida, United States of America; 5 Sugarcane Field Station, USDA-ARS, Canal Point, Florida, United States of America; 6 CIRAD, UMR BGPI, Montpellier, France, and BGPI, Univ Montpellier, CIRAD, INRAE, SupAgro, Montpellier, France; Universidade Federal de Lavras, BRAZIL

## Abstract

Sugarcane yellow leaf virus (SCYLV), the causal agent of yellow leaf disease, naturally infects at least three plant species in Florida: sugarcane (*Saccharum* spp.), the weed Columbus grass (*Sorghum almum*) and cultivated sorghum (*S*. *bicolor*). All three hosts are also colonized by the sugarcane aphid (*Melanaphis sacchari*), the main vector of SCYLV worldwide. To understand the high incidence of SCYLV observed in sugarcane commercial fields and in germplasm collections, we investigated the transmission efficiency of SCYLV from sugarcane and Columbus grass to sugarcane using the sugarcane aphid and a spider mite (*Oligonychus grypus*) that also tested positive for SCYLV in Florida. Healthy and SCYLV-infected leaf pieces of sugarcane and Columbus grass carrying viruliferous aphids or spider mites were transferred to virus-free plants of the yellow leaf susceptible sugarcane cultivar CP96-1252. Three- and 6-months post inoculation, the 108 aphid-inoculated plants of Columbus grass and the 90 mite-inoculated plants of sugarcane tested negative for SCYLV by tissue blot immunoassay (TBIA) or reverse transcription polymerase chain reaction (RT-PCR). Similar results were obtained for 162 aphid-inoculated plants of sugarcane, except for two plants that tested positive for SCYLV by TBIA and RT-PCR. In two field experiments planted with SCYLV-free and virus-infected sugarcane (cultivar CP96-1252), only 18–28% of healthy plants became infected during a 24- to 28-month period. SCYLV prevalence in these field experiments did not differ between aphicide treated and untreated plots. Incidence of *M*. *sacchari* haplotypes in the Everglades agricultural area also indicated that the predominant haplotype that is currently colonizing sugarcane was not a vector of SCYLV in Florida. Lack of virus transmission by the spider mite suggested that this arthropod only acquired the virus when feeding on infected plants but was unable to transmit SCYLV. The current vector of SCYLV in Florida remains to be identified.

## Introduction

The genus *Saccharum*, along with its related genera *Erianthus*, *Miscanthus*, and *Narenga* are all members of the *Poaceae* grass family. High sucrose content, useful by-products and ethanol, a bioenergy source, are all reasons why sugarcane (*Saccharum* interspecific hybrids) is an important economical asset to many tropical and sub-tropical areas around the world, including Florida. However, cultivating this crop holds its challenges. One of them is to control the numerous pathogens (including fungi, bacteria and viruses) that infect and cause diseases in sugarcane, subsequently resulting in yield losses [1]. Among the sugarcane infecting viruses occurring in Florida, sugarcane yellow leaf virus (SCYLV) is currently the most widespread and the one with highest effect on sugarcane production [2, 3, 4].

SCYLV is the causal agent of yellow leaf, a disease that was originally called yellow leaf syndrome of sugarcane or YLS. The disease was first reported in Hawaii in 1989 but there is evidence that the disease occurred in other locations much earlier [4, 5, 6]. Disease symptoms include intense yellowing of the leaf midrib while the leaf lamina remains green, and these symptoms were observed and first reported in central and eastern Africa in the 1960s and 1970s [5]. Since then, the disease has spread and is now found in most areas where sugarcane is cultivated [5, 7]. Over the years, research has focused on the impact of yellow leaf on sugarcane yields, as well as diversity and detection of the causal agent.

Yellow leaf can cause up to 25% yield losses in commercial fields, and a negative effect of SCYLV on sugarcane growth has been reported worldwide [5, 8, 9, 10, 11, 12, 13, 14]. In Florida, although most infected sugarcane cultivars are asymptomatic, yield losses ranging from 11 to 27% were reported in experimental fields [2, 15].

Worldwide isolates of SCYLV were recently distributed into two major phylogenetic clades and seven genotypes (BRA, CHN1, CUB, FLA1, FLA2, FLA3, and REU) [3]. Both genotypes BRA and CUB occur in the Everglades Agricultural Area (EAA) where sugarcane is produced in Florida [3, 15]. Although the biological significance of this genetic diversity is unknown, genotype CUB was more virulent than genotype BRA in controlled inoculation studies [16].

SCYLV is a member of the *Luteoviridae* family and cannot be transmitted mechanically. This virus is spread by infected stalk cuttings and by at least four aphid species in a persistent, circulative, and non-propagative manner: *Melanaphis sacchari*, *Ceratovacuna lanigera*, *Rhopalosiphum maidis* and *R*. *rufiabdominalis* [17, 18, 19]. Among these, *M*. *sacchari*, commonly known as the sugarcane aphid, is the most efficient vector of SCYLV worldwide and the most widespread in the Western hemisphere [5]. Despite the variability in transmission condition used, several studies have shown the ability and efficiency of *M*. *sacchari* to transmit SCYLV from infected to healthy sugarcane [16, 17, 18, 20, 21, 22, 23]. Though none of these experiments were conducted in Florida, the aphids used to transmit the virus in Scagliusi et al. [17] were supplied by the United States Department of Agriculture (USDA) Sugarcane Field Station in Canal Point, FL (BEL Lockhart, personal communication). Yellow leaf susceptible cultivar CP96-1252, which is grown on about a third of Florida’s commercial sugarcane production area [24], supports high numbers of *M*. *sacchari* in March-April in the EAA (Boukari and Rott, unpublished observations). These observations could explain the high incidence of SCYLV in commercial fields and germplasm collections in Florida [3]. Additionally, three genetically distinct haplotypes of the sugarcane aphid were recently reported in the USA: Haplotype H1 that mainly infests *Sorghum* species but also found on sugarcane, haplotype H3 that mainly colonizes sugarcane but also found on sorghum and Johnsongrass (*S*. *halepense*), and haplotype H6 that infests Johnsongrass and sugarcane [25].

Mites belong to the *Acari* order within the *Arachnida* class of arthropods. Among them, only members of the *Eriophyidae* and *Tenuipalpidae* families are known to transmit virus species of the *Fimoviridae*, *Potyviridae*, *Secoviridae*, *Alpha-* and *Betaflexiviridae* families [26]. The spider mite *Oligonychus stickneyi* has been present and occasionally a pest of sugarcane in Florida since the 1970s [27]. Another species, *Oligonychus grypus*, was found more recently on greenhouse plants in Canal Point and Belle Glade, FL [28]. These spider mites live and feed on the underside of sugarcane leaves, with infestations generally occurring between March and June [27]. Recently, spider mites collected from SCYLV-infected sugarcane tested positive for SCYLV by RT-PCR and sequencing [Sood et al. unpublished data]. This suggested that SCYLV might be vectored by spider mites, although *Luteoviridae* viruses are only known to be vectored by aphids [29].

Although SCYLV was successfully transmitted to several grass hosts under controlled conditions, sugarcane had been considered the only natural host of this virus for more than two decades [18, 30, 31, 32]. In 2014, the virus was reported in barley (*Hordeum vulgare*) in Tunisia [33]. More recently, SCYLV was found in both *Sorghum almum* and *S*. *bicolor* in Florida [34, 35]. The latter, commonly known as sorghum, is a grass species related to sugarcane and maize (*Zea mays*) which is not commercially grown in Florida. On the other hand, *S*. *almum*, also known as Columbus grass, is a robust, short-lived perennial grass that can be found worldwide between 25°N and 30°S latitudes and starting at sea level up to a 700 m altitude [36]. Columbus grass is considered one of the most valuable summer forage and fodder crops in semi-arid and sub-humid areas but also a noxious weed in several states of the USA and Australia [37, 38]. In Florida, this weed is copiously found growing year-round along the canals and near sugarcane fields in the EAA. Because Columbus grass is constantly growing near sugarcane, and because this grass is host to both *M*. *sacchari* and SCYLV, it is a potential reservoir for virus transmission to sugarcane.

The objectives of this study were to determine: 1) the efficacy of transmission of SCYLV from sugarcane to sugarcane using a known vector (*M*. *sacchari*) and a potential vector (*O*. *grypus*), 2) the efficacy of virus transmission from a “reservoir” plant (Columbus grass) to sugarcane using sugarcane aphids, 3) the SCYLV transmission capacity of field collected versus greenhouse raised sugarcane aphids, 4) the efficacy of an aphicide to limit the spread of SCYLV to healthy sugarcane under field conditions, and 5) the haplotypes of *M*. *sacchari* occurring on sugarcane and sorghum in Florida.

## Materials and methods

### Plant material for transmission assays

Virus-free plants of Columbus grass were grown in pots from seeds collected from field plants at Belle Glade, FL, and kept in a screened enclosure until aphid transfer. Virus-free sugarcane plants were obtained from disease-free single-bud cuttings from Kleentek^®^ stalks (http://certisusa.com/pest_management_products/tissue_culture_sugar_cane/kleentek.htm) or directly from tissue culture (Florida Crystals Corporation, Okeelanta, FL). These healthy plants were used for either rearing aphids or inoculation experiments. SCYLV-infected plants of Columbus grass were collected in the field at Belle Glade and transferred to pots in a greenhouse. Single-bud cuttings from SCYLV-infected stalks of sugarcane cultivars CL88-4730 (infected with genotype BRA) and CP88-1762 (infected with genotype CUB) were planted separately in pots in a greenhouse. Plants obtained from these cuttings were used for rearing viruliferous aphids. None of the plants infected by SCYLV showed symptoms of yellow leaf.

### Aphid rearing for SCYLV transmission

A colony of *M*. *sacchari* was collected in summer 2016 from a single sugarcane plant of cultivar CP96-1252 in the field at Belle Glade. Aphids were transferred on healthy leaf pieces (approx. 14 cm long) of the same cultivar in moist Ziploc bags at room temperature (~25°C). Aphids were moved in the laboratory to fresh virus-free sugarcane leaves every 3–4 days for 6 months to obtain a SCYLV-free colony of *M*. *sacchari*. Aphids were then moved onto healthy plants of Columbus grass in a growth chamber at 23°C and healthy plants of sugarcane cultivar CP96-1252 growing in an insect proof cage (61 x 61 x 142 cm, Bioquip, Rancho Dominguez, CA) in the greenhouse. Aphid colonies were kept on healthy Columbus grass and sugarcane plants until the beginning of the spring 2017. Aphids reared on Columbus grass were then moved onto caged SCYLV-infected plants of Columbus grass and aphids reared on sugarcane were moved onto caged SCYLV-infected sugarcane plants in the greenhouse to build up colonies of viruliferous *M*. *sacchari*.

### Aphid collection for SCYLV transmission without insect rearing

A field of sugarcane cultivar CP96-1252 with an incidence of SCYLV >90% was identified at Belle Glade in 2018. Plants of this field did not show symptoms of yellow leaf nor of any other known viral disease occurring in Florida such as mosaic, mild mosaic or leaf fleck. When the sugarcane field was colonized with *M*. *sacchari*, leaves carrying aphids were regularly collected from April 14 to June 22, 2018, cut in 3–5 cm sections, and immediately used in transmission assays as described below.

### Spider mites for SCYLV transmission to sugarcane

SCYLV-free plants of sugarcane cultivar CP04-1935 and both SCYLV-infected plants of sugarcane cultivars CL88-4730 and CP88-1762 in the greenhouse became infested with spider mites in early spring 2018. Leaves carrying mites were collected from these plants, cut in 12- to 15-cm long sections, and immediately used for transmission assays as described below.

### Detection of SCYLV in Columbus grass and sugarcane by tissue-blot immunoassay (TBIA)

Stalks of Columbus grass were cut transversally, and sections of the upper, middle, and lower part of each stalk were used to make imprints on nitrocellulose membranes. Similarly, duplicated imprints of collected sugarcane midrib cross-sections of leaves were made onto a nitrocellulose membrane. The membranes were then processed using SCYLV antibodies as described by Schenck et al. [39] and modified by Girard et al. [40].

### Detection of SCYLV in Columbus grass, sugarcane, aphids and mites by reverse transcription-polymerase chain reaction (RT-PCR)

Total RNA was extracted from sugarcane and Columbus grass leaves with the Qiagen RNeasy Plant mini kit (Qiagen, USA), and from mites (~100 pooled-mite samples) and aphids (10 pooled-aphid samples) with the Qiagen RNeasy mini kit (Qiagen, USA), following the manufacturer’s protocol. Arthropods were pooled before RNA extraction and molecular diagnosis in order to test a large number of individuals for presence or absence of the virus. Two experimental replicates (pooled samples) were used for aphids reared on virus-free Columbus grass, for aphids reared on virus-free sugarcane, and for spider mites collected from virus-free sugarcane. When arthropods were sampled from virus-infected materials, the number of experimental replicates was as follows: two for aphids reared on Columbus grass, four for spider mites collected from sugarcane, five for field-collected aphids from sugarcane, and 10 for aphids reared on sugarcane. Sample quality and presence or absence of SCYLV were determined by Reverse Transcriptase Polymerase Chain Reaction (RT-PCR) using Qiagen’s OneStep RT-PCR kit and ScYLVf1/ScYLVr1 primers as described by Girard et al. [40]. SCYLV amplicons (219 bp) were visualized under UV light on Sybr-green stained 1% agarose gels.

### Identification of SCYLV genotypes

To determine the genotype of SCYLV in infected plants, RNA samples that tested positive with diagnostic primers ScYLVf1/ScYLVr1 were then subjected to OneStep RT-PCR using genotype specific primers sets BRA-PER-F/BRA-PER-R and CUB-F/CUB-R as described by Abu Ahmad et al. [41]. The expected amplicons (362 bp for BRA-PER and 450 bp for CUB primers) were visualized under UV light on Sybr-green stained 1% agarose gels.

### Transmission assays

**Fig 1:**
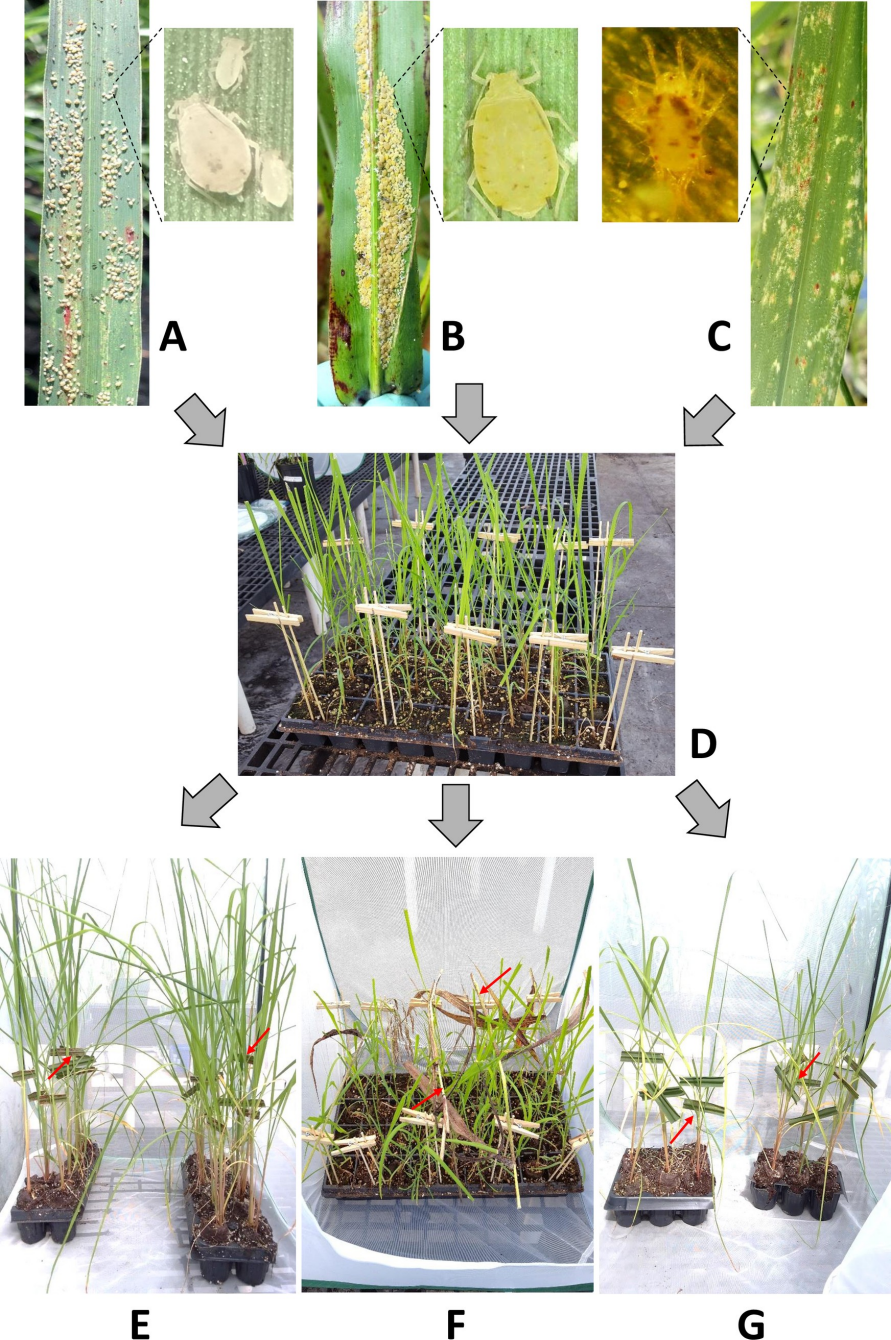
Sugarcane yellow leaf virus transmission steps. (A) SCYLV-infected sugarcane leaf with viruliferous *M*. *sacchari*. (B) SCYLV-infected leaf of Columbus grass with *M*. *sacchari*. (C) SCYLV-infected sugarcane leaf carrying the spider mite *Oligonychus grypus*. (D) Tray of virus-free CP96-1252 plantlets from tissue culture before virus transmission. (E) Infected sugarcane leaf fragments with aphids on caged CP96-1252 plantlets. (F) Infected leaf and/or stalk fragments of Columbus grass with aphids on caged CP96-1252 plantlets. (G) Infected sugarcane leaf fragments with spider mites on caged CP96-1252 plantlets.

For all assays, 1- to 2-month-old tissue culture (virus-free) plantlets of cultivar CP96-1252 were transplanted into trays and moved into insect-proof cages in the greenhouse for inoculation with viruliferous aphids or mites (Fig 1). Inoculated plantlets of all transmission experiments were then maintained in insect-proof cages for 14 days until arthropods were killed by application of an insecticide or a miticide. The insecticide to kill aphids was a granular pre-mix of 0.55% imidacloprid and 0.275% clothianidin (Tree & Shrub Protect and Feed Ready-To-Use Granules II, Bayer Advanced, USA). A teaspoon of the commercial product (approx. 6 g) was applied to the soil of each tray containing 50 plants (= approx. 33 mg of imidacloprid and 16.5 mg of clothianidin per tray). The miticide to kill spider mites was spiromesifen (Oberon 4SC, Bayer Crop Science, USA) mixed in water at 1.4 g of active ingredient per liter and applied on the plant foliage until run off with a handheld mist sprayer. Miticide application was performed twice in a 24 hr interval. Arthropods were dead within two to four days after application of the insecticide or miticide. Fourteen days after chemical application, the plantlets were removed from the insect-proof cages and transplanted into larger pots (3.8 liters) to allow growth in the greenhouse. Three and six months after inoculation, the top visible dewlap leaf (TVD) plus the second and third most developed (L+2 and L+3) leaves of each inoculated plant were used for TBIA as described above.

### SCYLV transmission from Columbus grass to sugarcane with aphids

Three plants of Columbus grass that tested positive for SCYLV by RT-PCR were transplanted into separate pots and moved to insect-proof cages. Colonies of aphids raised on healthy Columbus grass were then transferred to each infected Columbus grass plant for the aphids to acquire the virus on the SCYLV inoculum source. Aphid transfer on a single plant occurred on 24 April 2017, 15 May 2017, and 5 June 2017 for transmission experiments 1, 2, and 3, respectively, as described below. Reared aphids were allowed to probe and propagate on source plants for at least three weeks before transmission experiments were carried out. Twenty to 30 cm long pieces of SCYLV-free or infected leaves or stalks of Columbus grass were transferred onto virus-free 2-month-old plantlets of sugarcane cultivar CP96-1252 (Fig 1B & 1F). Each leaf piece carried 20–50 aphids. Three separate inoculation experiments were performed. A first set of 36 plantlets was inoculated with aphids raised on SCYLV-infected Columbus grass on 22 May 2017 (experiment 1). This was repeated on 13 June 2017 (experiment 2) and on 27 June 2017 (experiment 3). A total of 24, 24, and 18 plantlets were inoculated with aphids raised on virus-free Columbus grass on 23 May 2017 (experiment 1), 14 June 2017 (experiment 2), and 5 July 2017 (experiment 3), respectively. Experiment 3 also included 18 control plantlets that were not aphid-inoculated. Inoculated plantlets were handled and tested by TBIA as described above (Transmission assays). Incidence of SCYLV was expressed as the ratio of TBIA positive plants over the total number of inoculated plants. To confirm TBIA data and rule out false serological data, total RNA was extracted from randomly selected TVD leaves and processed by RT-PCR as described above. Results were expressed as the ratio of positive samples over the total number of tested samples.

### SCYLV transmission from sugarcane to sugarcane with aphids

These assays were performed in two different ways. First, colonies of *M*. *sacchari* raised on healthy CP96-1252 were transferred to genotype specific infected sugarcane plants (CL88-4730 for genotype BRA and CP88-1762 for genotype CUB) to build populations of viruliferous aphids. Sugarcane leaf fragments carrying 3–50 aphids reared on SCYLV free or infected plants were deposited on 2-month-old virus-free plantlets of sugarcane cultivar CP96-1252 (Fig 1A & 1E). Second, 3–5 cm leaf pieces with 3–10 aphids sourced from SCYLV-infected plants of CP96-1252 in the field were placed on healthy CP96-1252 plantlets from tissue culture. Trays of all inoculated plantlets were maintained in insect-proof cages in the greenhouse. Aphid-free plantlets of CP96-1252 maintained in insect-proof cages served as negative controls for both types of inoculation assays (reared or non-reared insects).

In a first experiment, 12 and 14 sugarcane plantlets were inoculated on 2 August 2017 with aphids raised on sugarcane infected with SCYLV genotype CUB and BRA, respectively. In a second experiment, 11 and 12 sugarcane plantlets were inoculated on 4 September 2017 with aphids raised on sugarcane infected with SCYLV genotype CUB and BRA, respectively. A subsequent experiment included healthy sugarcane (14 plantlets) inoculated with aphids collected from SCYLV-infected sugarcane in the field on 14 April 2018. This experiment was repeated on 16 April, 21 April, 27 April, 30 April, 15 May, 29 May, and 22 June 2018 using 24, 19, 17, 10, 8, 14, and 7 plantlets, respectively. The transmission experiment performed on 29 May 2018 also included 21 control plantlets that were not aphid-inoculated. The experiments were carried out and SCYLV incidence was determined by TBIA as described above (Transmission assays). SCYLV incidence was expressed as the ratio of TBIA positive plants over the total number of inoculated plants. Total RNA was extracted from either TBIA positive samples or randomly selected TVD leaves and processed by RT-PCR. The results were expressed as the ratio of positive samples over the total number of samples tested.

### SCYLV transmission from sugarcane to sugarcane with spider mites

Twelve to 15 cm leaf pieces from SCYLV-free (CP04-1935) or infected (CL88-4730 and CP88-1762) sugarcane plants harboring 100–1000 spider mites were transferred onto healthy trays of CP96-1252 plantlets maintained in insect-proof cages (Fig 1C & 1G). Caged plantlets of virus-free CP96-1252 without mites also served as negative control. Two separate inoculation experiments were performed, the first one on 13 March 2018 and the second one on 20 March 2018. In the first experiment, 25 plantlets were inoculated with mites collected from sugarcane cultivar CL88-4730 infected with SCYLV genotype BRA, and 25 plantlets were inoculated with mites collected from sugarcane cultivar CP88-1762 infected with SCYLV genotype CUB. This experiment also included 8 control plantlets that were not aphid-inoculated. In the second experiment, 20 and 20 plantlets were inoculated with mites collected from SCYLV-infected cultivars CL88-4730 and CP88-1762, respectively. This experiment also included 20 plantlets inoculated with mites collected from healthy sugarcane cultivar CP04-1935. The same timeline, parameters, detection methods and report format as the Columbus grass to sugarcane experiments were used for these experiments. RNA was extracted from randomly selected TVD leaves and processed by RT-PCR as described above.

### Determination of *M*. *sacchari* haplotypes

Aphids were collected from locations that were either public areas (no permit needed) or private sugarcane farms collaborating with the authors on numerous research and extension projects. Farm managers had given permission to collect aphids on their properties. A total of 140 aphids were collected in 2018–2019 from sugarcane (79 individuals) in Belle Glade, Okeelanta and Clewiston, FL; from sorghum (15 individuals) in Belle Glade; and from Columbus grass (46 individuals) at 10 locations in the EAA (S1 Table). Not more than two aphids were collected from the same plant and DNA was extracted from each single aphid using the Qiagen DNeasy Blood & Tissue kit according to the manufacturer’s protocol. The Cytochrome C oxidase subunit I (COI) gene used for genotyping was amplified by PCR as described by Nibouche et al. [25]. Amplification products (658 bp) were purified using the QIAquick Purification kit and sent to Eton Bioscience (North Carolina Branch) for bidirectional sequencing. Sequences were compared to the public sequences of haplotypes H1-H6 of *M*. *sacchari* in GenBank using blast search.

### Sugarcane experimental fields

SCYLV-free (healthy) and SCYLV-infected stalk cuttings of cultivar CP96-1252 were used to set up two trials on commercial sugarcane farms (Florida Crystals Corporation). One trial was established at Okeelanta on organic (muck) soil on 8 December 2016 and the other one was planted at 20 Mile Bend on mineral (sand) soil on 18 December 2016. At both experimental sites, a randomized block design with six blocks (replications) was used to compare plots planted with healthy (with or without insecticide applications) or virus-infected seed cane. The three treatments were randomized to plots that were 13.1 m long and six rows wide (1.5-m row spacing). All plots were surrounded on each side by a 4.6-m alley. Consecutive blocks were separated by same size blocks made of plots of cultivar CP01-1372 at Okeelanta and cultivar CPCL97-2730 at 20 Mile Bend. Each experimental field was 217 m long and 44.2 m wide.

### Insecticide applications in the sugarcane experimental fields

Flupyradifurone (Sivanto 200 SL, Bayer Crop Science, USA) was applied at 87.7 g of active ingredient per hectare to prevent aphid colonization in plots planted with healthy seed cane and treated with insecticide. A two-row boom backpack sprayer with 6 nozzles spaced 50.8 cm apart was used and calibrated to deliver 93.5 l/ha at 241.3 kPa of spray solution using a pressurized system with portable CO_2_ tanks. The insecticide was applied bi-weekly starting two to three months after planting or harvest. At Okeelanta, 12 applications were done in the plant cane crop and eight in the first ratoon crop. At 20 Mile Bend, nine applications were performed in the plant cane crop and seven in the first ratoon crop.

### SCYLV prevalence in the sugarcane experimental fields

The top visible dewlap leaf of 10 randomly selected sugarcane stalks per plot was collected at each of six different sampling dates after planting and over a 24- (20 Mile Bend) to 28-month (Okeelanta) period. Presence of SCYLV was tested by tissue blot immunoassay (TBIA) as described by Schenck et al. [39] and modified by Girard et al. [40]. SCYLV prevalence in each plot at each sampling date was expressed as the percentage of infection of 10 leaves.

### Statistical analysis

Data from the Okeelanta and 20 Mile Bend experiments were analyzed separately. For each experiment, SCYLV prevalence as affected by treatment and sampling date was analyzed using a linear mixed model (PROC GLIMMIX, SAS Institute Inc. [42]). Treatment, sampling date, and their two-way interaction were fixed effects, while block and block*treatment were random effects. Thus, the effect of repeated measures was modeled using a variance component covariance structure. Means were separated using the Tukey-Kramer adjustment [42].

## Results

### Detection of SCYLV in source plants harboring mites or used to rear aphids and in mites and aphids collected from these plants

SCYLV was not detected by TBIA or RT-PCR in any of the source plants used to rear healthy aphids or in healthy plants harboring mites. These plants included three plants of Columbus grass and two plants of sugarcane (cultivar CP96-1252) that were used for transmission assays with virus-free aphids and one sugarcane plant (cultivar CP04-1935) used for transmission assays with virus-free mites. SCYLV was detected by TBIA or RT-PCR in all the source plants used to rear viruliferous aphids for transmission assays: 3 plants of Columbus grass and 25 plants of sugarcane (21 field plants of cultivar CP92-1252, 2 greenhouse plants of cultivar CP88-1762 and 2 greenhouse plants of cultivar CL88-4730). The virus was also found in two sugarcane infected plants (cultivar CL88-4730) harboring mites.

Pooled aphid and mite samples collected from virus-free source plants all tested negative by RT-PCR: two aphid samples from Columbus grass, two aphid samples from sugarcane, and two mite samples from sugarcane. Aphids reared in the greenhouse tested positive for SCYLV when collected from virus-infected sugarcane (10 samples) and negative when collected from virus-infected Columbus grass (2 samples). Five aphid samples collected from virus-infected sugarcane plants in the field all tested positive for SCYLV by RT-PCR. Four mite samples collected from virus-infected plants in the greenhouse also tested positive for SCYLV by RT-PCR.

### Transmission of SCYLV from Columbus grass to sugarcane using aphids reared on virus-infected plants

Three separate SCYLV transmission experiments from Columbus grass to sugarcane were performed using aphids reared on healthy and infected plants. Three and six months after each inoculation, all plants (three leaves per plant) were tested for infection by SCYLV using TBIA. At both time points, the virus was not found in any of the 66 plants inoculated with aphids from virus-free Columbus grass plants or in the 18 non-inoculated plants (negative control plants). SCYLV was not detected either in a total of 108 plants inoculated with aphids reared on virus-infected plants. Fourteen leaf samples randomly collected across the three experiments also tested negative for the virus by RT-PCR: 8 plants inoculated with aphids raised on SCYLV-infected Columbus grass, 4 plants inoculated with aphids raised on SCYLV-free Columbus grass, and 2 plants not inoculated with aphids.

### Transmission of SCYLV from sugarcane to sugarcane using aphid reared on virus-infected plants

Two separate SCYLV transmission experiments from sugarcane to sugarcane were performed using aphids reared on plants infected by two different genotypes of the virus. Inoculated plants were tested for virus infection by TBIA three and six months after each inoculation, using three leaves per plant. Three months after inoculation, SCYLV was not found in the 23 plants (total of both experiments) inoculated with aphids from sugarcane plants infected by SCYLV genotype CUB. Six months after inoculation, two of these 23 plants tested positive for SCYLV by TBIA and RT-PCR. These two plants were both from the set of 12 plants inoculated on 2 August 2017. The 26 plants inoculated with aphids collected from sugarcane infected by genotype BRA tested negative by TBIA at both time points. Eight leaf samples collected across the two experiments and that tested negative by TBIA also tested negative for the virus by RT-PCR.

### Transmission of SCYLV from sugarcane to sugarcane using aphids collected in the field

Eight separate transmission experiments from sugarcane to sugarcane were performed with viruliferous aphids collected in the field and transferred immediately on healthy sugarcane plants in the greenhouse. Three and six months after inoculation, the 113 plants inoculated with viruliferous aphids and 21 negative control plants all tested negative for SCYLV by TBIA. Sixteen randomly selected samples from inoculated plants and two from control plants also tested negative for the virus by RT-PCR.

### Transmission of SCYLV from sugarcane to sugarcane using mites collected from virus-infected sugarcane

Two separate SCYLV transmission experiments from sugarcane to sugarcane were performed with viruliferous mites. Inoculated plants (three leaves per plant) were tested by TBIA three and six months after inoculation, and results were identical at both time points: SCYLV was not detected in any of the 90 plants inoculated with mites collected from plants infected with either genotype BRA (45 plants) or CUB (45 plants). The 20 plants inoculated with mites from virus-free sugarcane leaves and the eight non-inoculated control plants also tested negative for SCYLV by TBIA. The virus was not detected either by RT-PCR in 12 leaf samples collected randomly across the two experiments.

### Haplotypes of *Melanaphis sacchari*

All 61 aphids collected from Columbus grass and sorghum belonged to haplotype one (H1) of *M*. *sacchari* (Table 1). Haplotypes one, two, and three (H1, H2, H3) were found among the 79 aphids collected on sugarcane. H3 was the most abundant haplotype (88.6% of tested aphids), followed by H2 (10.1%) and H1 (1.3%) (Table 1). Four aphid samples that were collected from SCYLV-infected sugarcane plants in the field and used for transmission studies all belonged to haplotype H3.

**Table 1:** Frequency of *Melanaphis sacchari* haplotypes occurring on three host plants in the Everglades Agricultural area.

	Frequency (%) of *M*. *sacchari* haplotype^a^		
Host	H1	H2	H3
Sugarcane	1.3 (1/79)^b^	10.1 (8/79)	88.6 (70/79)
Columbus grass	100 (46/46)	0 (0/46)	0 (0/46)
Sorghum	100 (15/15)	0 (0/15)	0 (0/15)

^a^ Determined as reported by Nibouche et al. [25]

^b^ Numbers in parentheses are the number of aphids belonging to the haplotype over the total number of aphids tested

### Field infection of healthy sugarcane by SCYLV and efficacy of insecticide applications

**Fig 2:**
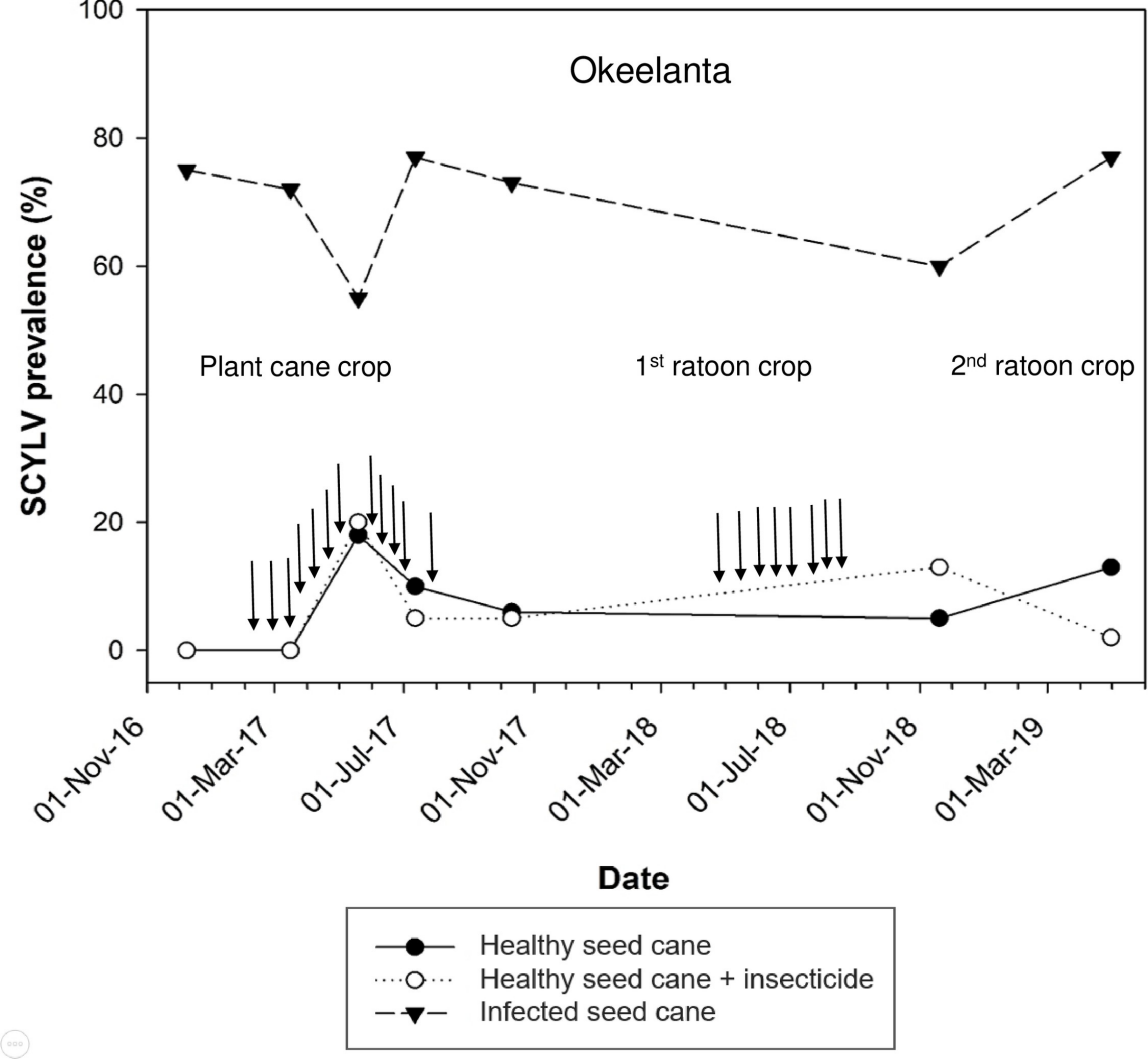
Progress of prevalence of sugarcane yellow leaf virus (SCYLV) in sugarcane cultivar CP96-1252 grown on organic soil (at Okeelanta). Sugarcane was planted on 8 December 2016 and harvested at the end of the plant cane crop on 28 January 2018. Sugarcane was harvested in the first ratoon crop on 2 December 2018. Prevalence of SCYLV was determined from December 2016 to May 2019. Insecticide was applied in the plant cane crop at the following dates (vertical arrows): 13 February 2017, 28 February 2017, 16 March 2017, 29 March 2017, 12 April 2017, 26 April 2017, 9 May 2017, 23 May 2017, 6 June 2017, 20 June 2017, 5 July 2017 and 19 July 2017. Insecticide applications were performed in the first ratoon crop at the following dates (vertical arrows): 2 May 2018, 21 May 2018, 4 June 2018, 19 June 2018, 7 July 2018, 31 July 2018, 16 August 2018 and 29 August 2018. Each data point represents the mean percent of infection of six plots (10 leaves/plot).

**Fig 3:**
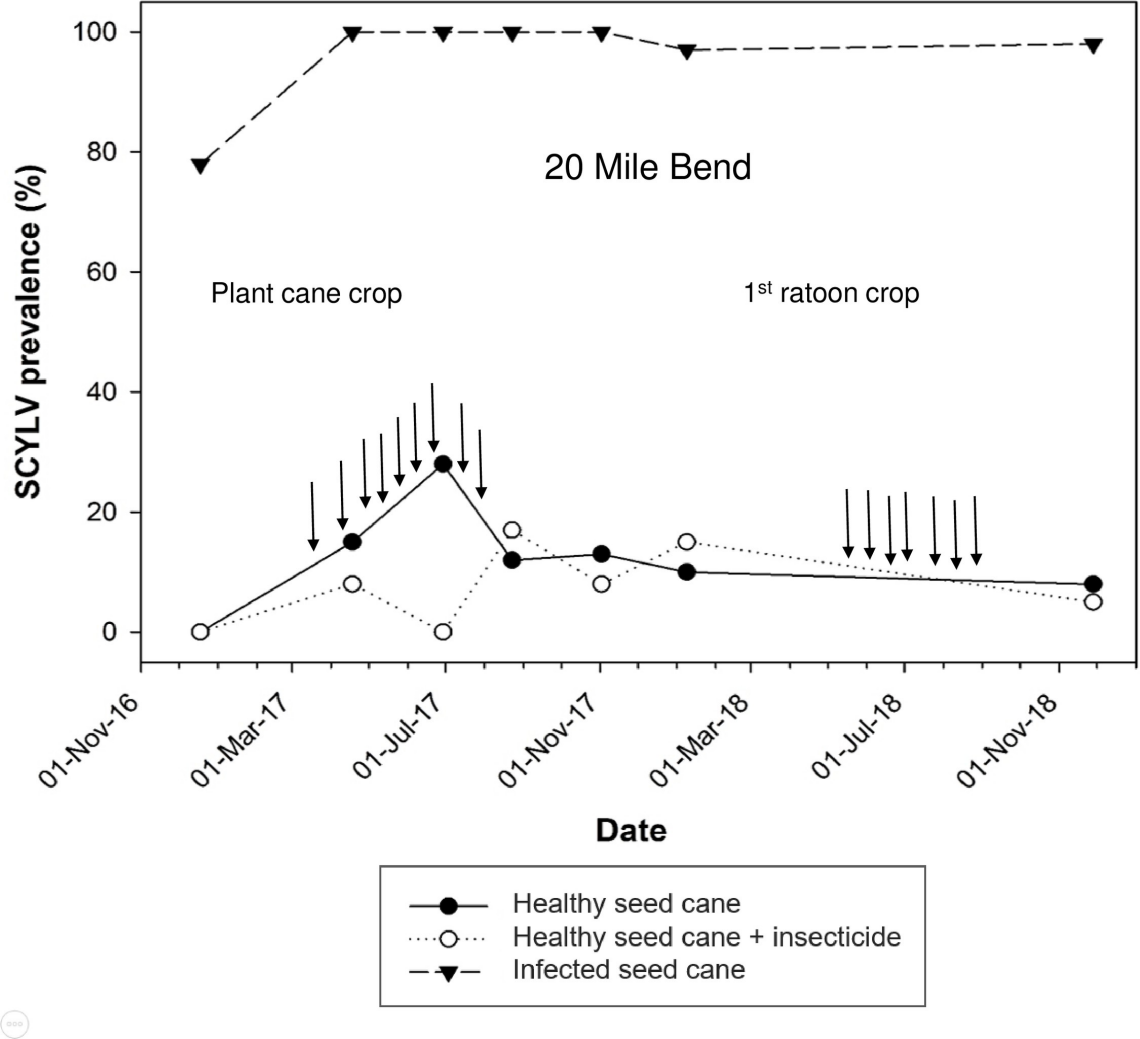
Progress of prevalence of sugarcane yellow leaf virus (SCYLV) in sugarcane cultivar CP96-1252 grown on mineral soil (at 20 Mile Bend). Sugarcane was planted on 18 December 2016 and harvested at the end of the plant cane crop on 17 March 2018. Sugarcane was harvested in the first ratoon crop on 13 February 2019. Prevalence of SCYLV was determined from December 2016 to the end of November 2018. Insecticide was applied in the plant cane crop at the following dates (vertical arrows): 21 March 2017, 19 April 2017, 3 May 2017, 17 May 2017, 31 May 2017, 14 June 2017, 28 June 2017, 12 July 2017 and 26 July 2017. Insecticide applications were performed in the first ratoon crop at the following dates (vertical arrows): 22 May 2017, 4 June 2018, 20 June 2018, 7 July 2018, 31 July 2018, 17 August 2018 and 31 August 2018. Each data point represents the mean percent of infection of six plots (10 leaves/plot).

From December 2016 to April 2019, SCYLV prevalence in infected plots of cultivar CP92-1252 planted on organic soil at Okeelanta varied from 55% to 77% (Fig 2). Prevalence of the virus in infected plots of the same cultivar planted on mineral soil (20 Mile Bend) ranged from 78 to 100% during December 2016 to November 2018 (Fig 3). Plots of cultivar CP96-1252 planted with healthy seed cane became infected with SCYLV within a few months after planting in both experimental locations and in both insecticide-treated and non-treated sugarcane. The highest prevalence of SCYLV was observed at Okeelanta in the plant cane crop: 20% for plots treated with the insecticide and 18% for untreated plots (Fig 2). At 20 Mile Bend, the highest prevalence of the virus was also found in the plant cane crop: 28% for untreated plots and 17% for plots sprayed with the insecticide (Fig 3). Over the entire duration of the experiments, a virus-infection effect was observed at both locations between plots planted with infected seed cane and plots planted with healthy seed cane (Table 2). No sampling date (F = 0.58; df = 5, 75; P = 0.715) and no treatment by sampling date effect (F = 1.6; df = 10, 75; P = 0.130) were observed at Okeelanta. The same result was obtained for sampling date at 20 Mile Bend (F = 0.91; df = 5, 75; P = 0.483) but a treatment by sampling date interaction was observed for this location between infected plots and insecticide treated or untreated healthy plots of cultivar CP96-1252 (F = 2.9; df = 10, 75; P = 0.004). There was no significant difference in virus prevalence between untreated healthy sugarcane and insecticide treated healthy sugarcane at either location (Table 2). No yellow leaf symptoms were observed on sugarcane plants infected by SCYLV, regardless of crop season, treatment and location.

**Table 2:** Analysis of sugarcane yellow leaf virus (SCYLV) prevalence in sugarcane plots planted with healthy and infected CP96-1252 seed cane and grown from December 2016 to May 2019 at Okeelanta and from December 2016 to November 2018 at 20 Mile Bend.

Okeelanta (organic soil)		20 Mile Bend (mineral soil)	
Treatment	Mean SCYLV prevalence (%)^a^	Treatment	Mean SCYLV prevalence (%)^a^
Healthy seed cane	8.9 b	Healthy seed cane	14.4 b
Healthy seed cane + insecticide	7.5 b	Healthy seed cane + insecticide	8.9 b
Infected seed cane	69.2 a	Infected seed cane	98.9 a
F value	158.0	F value	186.7
df	2, 10	df	2, 10
P > F	< 0.001	P > F	< 0.001

^a^ Means in the same column followed by the same letter are not significantly different (Tukey-Kramer adjustment, *α* = 0.05). Statistical analysis was performed with all prevalence data (see Figs 2 and 3).

## Discussion

In this study, transmission assays using sugarcane aphids collected from SCYLV-infected leaves resulted in nearly no infection of inoculated plants (only two positive plants of a total of 270 inoculated plants). This is the first report of failure of transmission of SCYLV using *M*. *sacchari* while ensuring the presence of SCYLV in source plants (Columbus grass and sugarcane) and aphids (from sugarcane). Several studies are available in the literature reporting from 29–100% transmission efficiency of SCYLV using this aphid species (Table 3). In successful aphid transmission of SCYLV, the virus was detectable in inoculated plants as early as seven days to three weeks after inoculation [20, 21]. The entire green top of the sugarcane plant can be colonized by the virus within 9–11 weeks after inoculation [21]. In our study, the virus remained undetectable in the top three leaves three and six months after inoculation.

**Table 3:** Characteristics of published transmission experiments from sugarcane (*Saccharum* spp.) infected by sugarcane yellow leaf virus (SCYLV) to healthy sugarcane with the aphid *Melanaphis sacchari*.

Inoculated sugarcane variety	Number of *M*. *sacchari*	Feeding time on SCYLV-infected plants	Inoculation access period	Transmission efficiency period	Transmission efficiency (%)	Insecticide used to eliminate aphids	Application type	Insecticide type	Reference
CP65-357	20–30	48–72 h	≥ 12 h	12 months	100	Not specified^a^	-	-	Scagliusi and Lockhart [17]
H73-6110 H87-4094	4–100	48 h	≥ 3 d	2–6 months	100	None	-	-	Schenck and Lehrer [18]
H87-4094	10	NA	NA	4 weeks	89	None	-	-	Schenck et al. [23]
H87-4094	10–200	NA	7 d	3–11 weeks	NA	None	-	-	Lehrer et al. [21]
CC84-75 CC89-68 SP71-6163	7 to 10 (nymphs)	3–5 weeks	48–72 h	5–6 months	29–71	Imidacloprid at 100 ppm	Foliar spray	Systemic	Abu Ahmad et al. [16]
R570	5 to 30	≥ 2 weeks	2, 17, and 28 d	45 d	100	Thiamethoxam at 62.5 mg/l	Foliar and soil drenching	Systemic	Behary Paray et al. [22]
Co86-032	5	1 month	7 d	7 d	100	Malathion (5 ml/l of water)	Foliar spray	Non-systemic, broad spectrum	Chinnaraja and Viswanathan [20]
CP96-1252	3 to 50	≥ 3 weeks	2 weeks	3–6 months	0–0.7	Imidacloprid (33 mg) and clothianidin (17.5 mg) per 3.6 l pot	Granules applied to soil	Systemic	This study

^a^ Not specified = an insecticide was used but no information given regarding product, active ingredient or dosage.

NA = not available.

Virus transmission can be affected by several factors such as plant age, the number of aphids used for transmission, the feeding time and the inoculation access period. In our study, we used young tissue cultured plantlets similar to those reported for successful transmission of SCYLV by Chinnaraja and Viswanathan [20]. In preliminary studies (data not shown), we also tested different inoculation access periods from 2–7 days. No transmission was obtained with these access periods although they were successfully used by others (Table 3). The two-week access period used in this study was therefore much longer than the ones reported so far, which should have facilitated or increased virus transmission. The number of aphids (up to 50 aphids) used in our study for transmission of the virus was also similar or higher than in other reported studies (Table 3).

Although this is the first SCYLV transmission study done in Florida, these findings were unexpected because aphids originating from Florida (Canal Point) were used to demonstrate that *M*. *sacchari* was an efficient insect vector of SCYLV [17]. On the other hand, this latter study was conducted 20 years ago, and several aspects of disease epidemiology have changed since then. Sugarcane cultivars grown in the EAA in the 1990s are no longer grown commercially and new natural hosts of SCYLV have recently been identified [34, 35]. Furthermore, in 2013, massive outbreaks of *M*. *sacchari* occurred on sorghum in the USA [43]. These outbreaks were attributed to the invasion of sorghum in the Americas by a new superclone of this aphid [25]. This dominant superclone was identified as a specific clonal lineage (MLL-F) belonging to haplotype H1 of *M*. *sacchari* [25]. Invasion of sorghum by a new clone of the sugarcane aphid resulted in a change of the population structure of *M*. *sacchari* in the USA. In Louisiana, aphids collected on sugarcane and Johnsongrass in 2007 all belonged to lineage MLL-D or haplotype H3 whereas haplotypes H1 and H3 were found on sugarcane in 2013–2017 [25]. In a survey performed in the early 2000s in Louisiana, incidence of SCYLV in fields planted with virus-free seed cane varied only between 2–5% over a 21 months period, suggesting low spread of the virus in this geographical location [44].

The haplotypes of *M*. *sacchari* present in Florida before the 2013 aphid outbreaks on sorghum are unknown. Aphids collected in this study in 2018 and 2019 belonged to three haplotypes (H1, H2, and H3). Only haplotype H1 aphids were found on sorghum species (*S*. *bicolor* and *S*. *almum*) whereas aphids of haplotype H1, H2, and H3 were all three collected from sugarcane. Nevertheless, H1 aphids were rarely found on sugarcane (only one of 79 samples) and most aphids (>88%) belonged to haplotype H3 which was used in our transmission assays and failed to spread SCYLV from infected to healthy plants. The third haplotype (H2) was found at low frequency (10%) and its presence in Florida is reported here for the first time. Interestingly, haplotype H2 is the only one so far reported in Guadeloupe (French West Indies) where SCYLV has been efficiently transmitted from infected to healthy sugarcane plants under controlled conditions [16, 25]. These data suggest that not all haplotypes of *M*. *sacchari* are able to transmit SCYLV and the capacity of H1 and H2 aphids to transmit SCYLV to sugarcane in Florida has yet to be explored.

Columbus grass has been identified as a secondary host of SCYLV and *M*. *sacchari* only recently, and this is the first reported attempt to use Columbus grass in virus transmission studies. In contrast to aphids collected from infected sugarcane, SCYLV was not found in aphids reared on infected Columbus grass plants. SCYLV may have not been found in aphids raised on Columbus grass because the virus titer was low in this plant species or because the aphid haplotype that was used for these transmission experiments originated from sugarcane and was not adapted for transmission to sorghum species. Previous studies showed that the virus can be detected in *M*. *sacchari* (most likely haplotype H1) collected from SCYLV-infected plants of *S*. *bicolor* in Florida [35].

Inefficient spread of SCYLV by haplotype H3 from sugarcane to sugarcane could be caused by several factors such as mutations in the virus affecting its transmission efficiency or by a modification of aphid transmission capacity. *Buchnera aphidicola*, a symbiotic bacterium of aphid vectors, is involved in *Luteoviridae* virion transmission [29]. Luteovirids’ transmission can be influenced by the insect genotype and symbiont-genotype interaction, as well as by a *Buchnera*-regulated plant defense against aphids or the synthesis by *Buchnera* of a chaperonin that facilitates the virus movement in the aphid hemolymph [45, 46, 47, 48]. Differences in capacity of transmission between haplotypes of *M*. *sacchari* may occur in Florida and the lack of *Buchnera* symbiont may result in incapacity to acquire/transmit SCYLV. Furthermore, insects (and aphids in particular), can also induce a range of plant responses associated with pathogen resistance instead of insect resistance [49]. A locus in melon (*Cucumis melo*), the Vat gene, reduces both aphid feeding and virus transmission [50, 51]. On the other hand, the systemic insecticide used in our experiments to eliminate aphids after inoculation contains imidacloprid that is known to induce plant defenses and trigger RNA silencing which serves as an antiviral and antibacterial defense system [52, 53, 54]. However, imidacloprid applied as foliar spray (soil application in our study) did apparently not interfere with transmission of SCYLV under controlled condition in Guadeloupe [16].

Although source plants used in our study for virus transmission did not show any virus disease symptoms, it cannot be excluded that these plants were infected by a cryptic virus or another microorganism that interacted with SCYLV, thus preventing virus transmission. Further investigations need to be undertaken to test this hypothesis.

In two separate field experiments conducted in this study, sugarcane that was healthy at planting became infected by SCYLV. However, prevalence of the virus was low (maximum 28%) during two crop seasons (plant cane and first ratoon crop) although healthy plots of cultivar CP96-1252 were planted in proximity to infected plots. Fluctuations of SCYLV prevalence during these two crop seasons, especially the apparent disappearance of the virus at some time points could be ascribed to sample size (10 stalks out of several hundred per plot) or to titer variations at different growth stage of the sugarcane plant (low and undetectable virus concentrations). The latter is often associated with luteovirids and virus detection that was impeded using serological methods such as TBIA [55, 56, 57]. No significant difference in SCYLV prevalence was found at Okeelanta and 20 Mile Bend between untreated healthy plots and healthy plots treated with the insecticide flupyradifurone, which has strong aphicidal properties [58]. Besides controlled transmission data, these field data also suggested that *M*. *sacchari* was currently not, if at all, efficiently spreading SCYLV in Florida sugarcane fields. Consequently, other vectors might be involved in infection of sugarcane by this virus.

Spider mites have been occasional pests of sugarcane in Florida. Unlike luteoviral aphid vectors that use their long stylets to probe and directly feed from the phloem, these mites use their sharp stylets to penetrate and remove the cell contents from plants. Although SCYLV was systematically detected in spider mite samples collected from virus-infected sugarcane, it was never found in inoculated plants, thus suggesting that this arthropod only acquired the virus while feeding on infected plants but was unable to transmit SCYLV. Future research needs to focus on other possible arthropod vectors of the virus, as well as transmission efficiency of all known haplotypes of *M*. *sacchari*.

## Supporting information

S1 Table Characteristics of *Melanaphis sacchari* samples collected in the Everglades Agricultural Area for determination of aphid haplotypes. (XLSX)
